# Dragon Kill Points: applying a transparent working template to relieve authorship stress

**DOI:** 10.1186/s12915-026-02521-x

**Published:** 2026-01-29

**Authors:** April Robin Martinig, Spenser L. P. Burk, Szymon M. Drobniak, Isabella Perry, Kyle Morrison, Megan Petersohn, Patrice Pottier, Shinichi Nakagawa, Pietro Pollo, Lorenzo Ricolfi, Coralie Williams, Ayumi Mizuno, Aimee Chhen, Jesse Tam, Yefeng Yang, Julia de Jong, Alberto Ceccacci, Sandra Cuadros, Malgorzata Lagisz

**Affiliations:** 1 The Okanagan Institute for Biodiversity, Resilience, and Ecosystem Services, University of British Columbia, Kelowna, BC Canada; 2 Départment de biologie, Université de Sherbrooke, Sherbrooke, Canada; 3 Evolution & Ecology Centre and School of Biological, Earth and Environmental Sciences, University of New South Wales, Sydney, Australia; 4 Unaffiliated, Calgary, Canada; 5 Institute of Environmental Sciences, Faculty of Biology, Jagiellonian University, Kraków, Poland; 6 Department of Biological Sciences, University of Alberta, Edmonton, Canada; 7 Department of Biology, Faculty of Science, Hokkaido University, Hokkaido, Japan; 8 Department of English, University of Calgary, Calgary, Canada; 9 Department of Economics, Philosophy and Political Science, I.K. Barber Faculty of Arts and Social Sciences, The University of British Columbia, Kelowna, BC Canada

**Keywords:** Accountability, Coauthorship, Collaborative, Credit, Publishing, Contributorship

## Abstract

Classifying and ranking academic authorship lists is complex in practice, despite existing frameworks, and can lead to conflict. We propose Dragon Kill Points, adapted from multiplayer gaming, to track contributions throughout a project’s lifecycle. Dragon Kill Points is based on five principles: granularity, responsibility, equity, autonomy, and transparency (GREAT). These ensure detailed task records, clear criteria, equitable rules, contributor flexibility, and shared documentation. By applying Dragon Kill Points, teams can reduce disputes, promote inclusivity, and recognise all contributions, including middle authorship. This scalable system offers a practical solution for managing authorship in collaborative research.

**Supplementary Information:**

The online version contains supplementary material available at 10.1186/s12915-026-02521-x.

## From quests to credits: rethinking authorship

Acknowledgement is the greatest form of currency in the realm of human connection.

- Anonymous

**Fig. 1:**
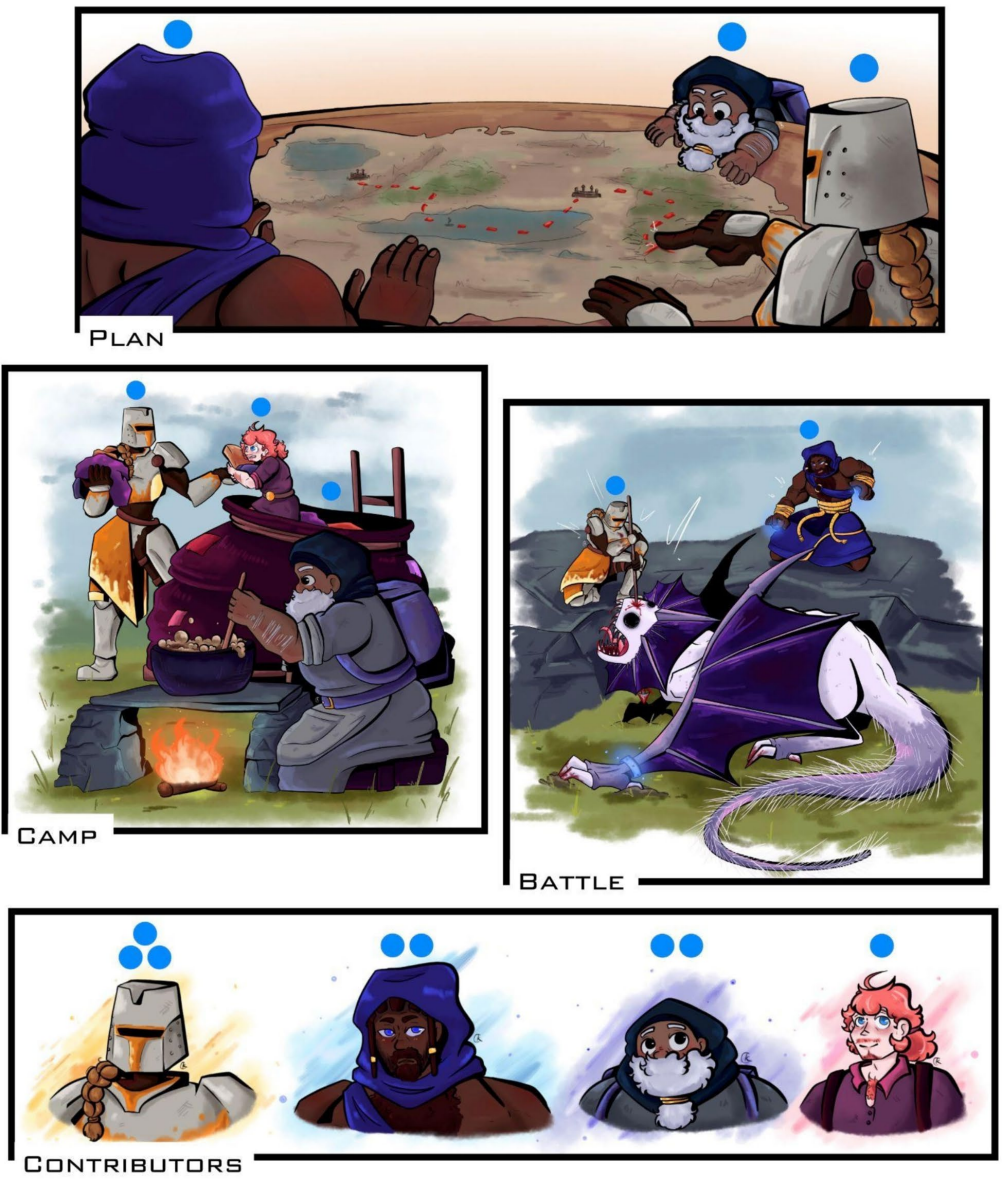
An imaginary quest with four party members. Not all party members contribute to all parts of the quest. Top panel: planning the quest (analogous to “conceptualization”). Left panel: setting up camp (analogous to “data collection”). Right panel: battling the dragon (analogous to “writing the manuscript”). Bottom panel: Party members (contributors) are shown from highest contribution (first) to lowest contribution (last). Final authorship order can vary depending on discipline. Blue circles are used to track contributions

Research is like a quest to slay a dragon (Fig. 1; Glossary). Despite common folklore, you seldom go on this quest alone. But, when your team slays the dragon, how do you decide who deserves to reap the rewards? Is it just the person who delivered the final blow, or should others who contributed to the effort also be recognised—and how are different levels and types of contributions accounted for? These contentious considerations can lead to internal party frictions along the way, while in a research context, these same dilemmas can lead to authorship disputes [1–4]. These disputes can arise both over who qualifies as an author at all and over the order in which authors are listed. Much of the stress around authorship arises from determining how each person contributed, assessing whether those contributions qualify them as authors, and then deciding how these contributions translate to authorship order.

Authorship order is a common way to reward contributions (e.g., [5–7]). For example, in biology, earlier authorship positions are valued more than later positions, apart from the last author. However, when it comes to evaluating a researcher’s “impact”, not all positions are given the same value [6–12]. As a result, authorship order is an increasingly contentious issue with the rise of multi-authored papers and the corresponding fall of single-authored ones [13–18]. Ideally, authorship order should reflect contributions in line with the conventions of a given field [6, 11, 19, 20]. However, you would be hard-pressed to find someone who has not been burned by this assumption—whether by believing their contributions deserved a higher position on the authors list or by feeling their efforts went unacknowledged [15, 21–24].

Formal frameworks to acknowledge contributions have been developed, used, discarded, ignored, reinvented, and improved (e.g., [11, 25–51]). While these frameworks are a vast improvement over not acknowledging author contributions at all, they are implemented after the project is completed, being used to justify, rather than create, the authorship list. We present a solution to this and several other problems using an idea borrowed from multiplayer gaming [52]: tracking Dragon Kill Points from the start of the project to translate contributions into authorship positions for the modern day dragonslayers.

**Table:** 

Dragon Kill Points Glossary	
**Dragon**: A metaphor for the challenges or objectives tackled in a project. This could represent anything from solving a problem to conducting a large-scale experiment or finishing a final product	**Loot**: The tangible and intangible outcomes of a project, including rewards or recognition gained. Examples include the knowledge generated, the impact of the project, professional opportunities, or recognition within the community
**Dragonslayer**: An individual contributor to the project. This can be anyone who contributes to the project in any way	**Party**: The collaborative team working together on a project. It includes all individuals involved, regardless of their specific roles or contributions
**Dragon Kill Points**: A system to track participation during quests that ensures a fair distribution of loot, which can then be spent on rewards. Here it is adopted to track and quantify contributions throughout a project's lifecycle	**Quest**: The overall endeavour or goal that the team is working towards

## The problem with the current status quo

Quests to slay the dragons of today may no longer resemble those of mythology, but they still hold the power to transform lives through the records kept, the reputations built, and the loot divided. But what if the loot distribution is based on how shiny each dragonslayer’s armour appears before the quest starts, rather than their actual deeds?

In some realms, such as economics and political science, loot is still divided by alphabetical order—a tradition meant to avoid disputes and promote fairness [13, 53, 54]. While Einav and Yariv [55] found that alphabetical ordering can have measurable effects on career success for some authors, and Joannis and Patil [56] found alphabetical ordering increased article quality while disincentivising teamwork, Abramo and D’Angelo [57] found little to no evidence of systematic surname effects on individual-level citability. Its supposed benefits, such as increased article visibility, are minimal [58], and the approach is increasingly ill-suited as multi-authored papers become more common [36, 47, 59, 60].

Whether through pre-set hierarchies or unspoken rules, systems that ignore the scale and nature of each contributor’s efforts risk perpetuating inequities. Thus, what if there was no agreement beforehand on what qualifies someone as a dragonslayer, and the rules are only created after the dragon has been slain? This may sound outlandish, but it parallels what modern-day dragonslayers face when it comes to academic authorship [61].

### The current systems lack granularity (G)

**Fig. 2:**
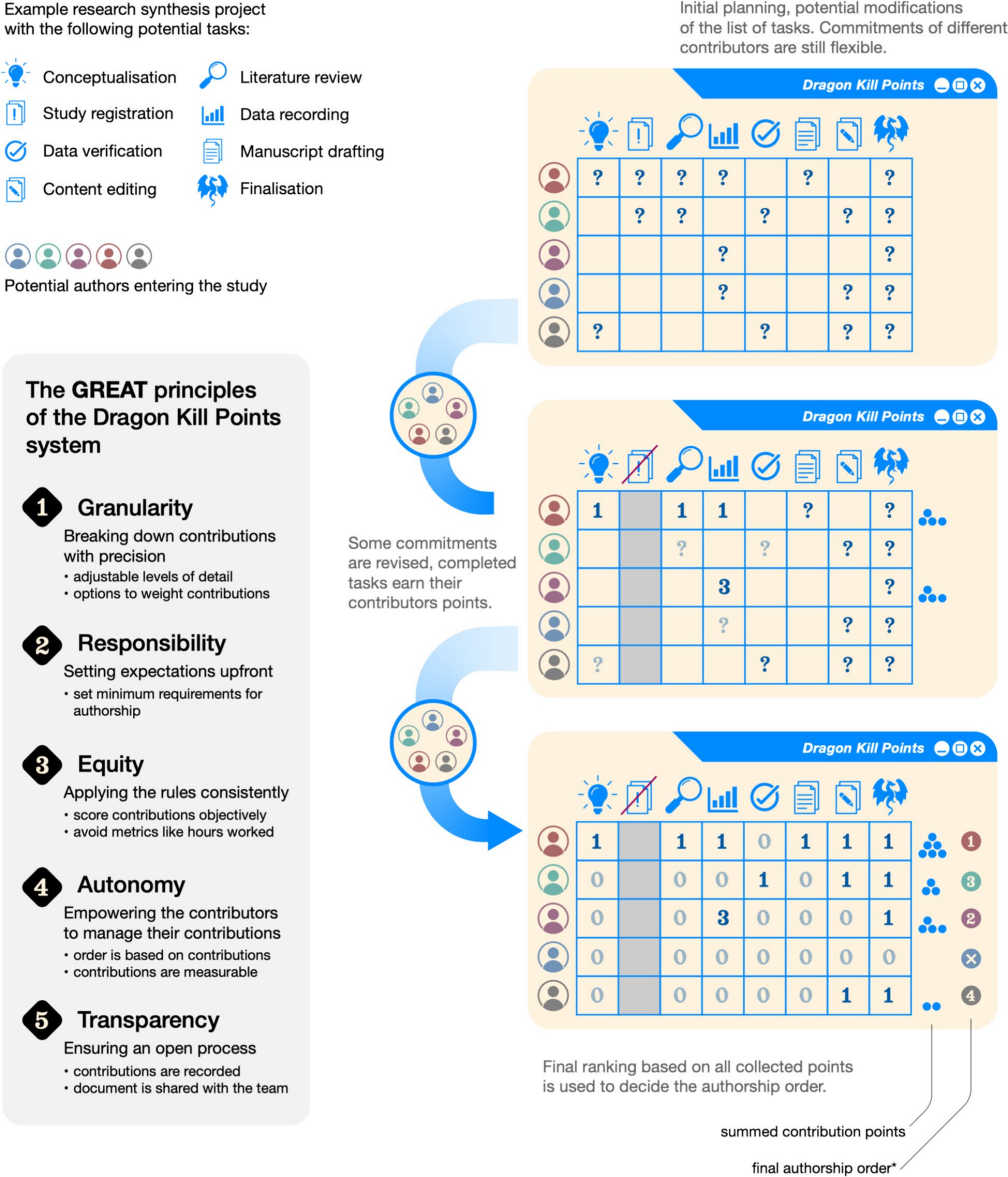
Visualisation of the steps involved with implementing the Dragon Kill Points alongside the GREAT principles. Asterisk indicates final authorship order can vary depending on discipline

If contributions are only recorded in broad categories (e.g., directly fighting the dragon, keeping watch, and setting up camp), we lose sight of each dragonslayer’s specific efforts (e.g., performing these roles dutifully every day for 100 days versus once). In research, Contribution Roles Taxonomy (CRediT) consists of 14 broad roles (categories summarised in Table 1; [35, 38, 62]) with the optional specification of the degree of contribution (as lead, equal, or supporting; [63, 64]). However, the specification of the degree of contribution is seldom used (but see [11]) and remains a major shortcoming of how authorship contribution statements are currently written [22, 43, 49, 65–68]. New higher-resolution systems like Method Reporting with Initials for Transparency (MeRIT) appeal to this type of granularity because they allow authors’ initials to be included alongside specific tasks within the manuscript itself [50]. MeRIT, however, is restricted to the methods section and does not capture a full range of contributions. Quests are comprised of multiple parts, not just the final act of slaying the dragon (Fig. 2). Research is no different.

**Table 1:** Potential tasks within categories used in the implementation of Dragon Kill Points. Categories and tasks may be added or removed as relevant, and categories can be broken down into more specific tasks for the contribution tracking template depending on project needs, location (e.g., field site, country), or timing (e.g., year). Contributions are recorded regardless of authorship status to ensure appropriate acknowledgement in research outputs. For an exhaustive, open-source, community-driven list of contribution categories, see the Contributor Role Ontology (https://data2health.github.io/contributor-role-ontology/). In brief, the Contributor Role Ontology is a vocabulary of roles, while Dragon Kill Points is a system for quantifying and applying those roles to determine authorship order

Category name	Description	Examples of tasks
Funding^a^	Obtaining and managing financial resources necessary to initiate and sustain the project. This includes securing funds, budgeting, and resource allocation to support all project activities^a^	- Research funding opportunities - Grant proposal writing - Budget planning - Contract negotiation - Financial management - Financial reporting - Financial compliance assurance
Conceptualisation^a^	Developing and defining the core ideas, hypotheses, and objectives that form the foundation of the research project. This involves formulating research questions, theoretical frameworks, and overall project goals^a^	- Literature review - Research question formulation - Hypothesis development - Objective setting - Theoretical framework construction - Collaborative ideation - Methodological conceptualisation
Project Administration^a^	Overseeing the organisational, logistical, and administrative tasks to ensure the project progresses efficiently and adheres to timelines and regulations. This includes planning, coordination, regulatory compliance, and risk management^a^	- Project scheduling - Meeting coordination - Documentation management - Progress tracking - Communication facilitation - Regulatory compliance - Risk management
Team Assembly and Training^b^	Recruiting and organising a team with the necessary expertise, and providing training to enhance their skills relevant to the project. This ensures that all team members are prepared to contribute effectively^b^	- Role definition - Recruitment processes - Onboarding sessions - Training workshops - Team-building activities - Role assignment - Professional development
Investigation^a^	Performing background tasks related to research and data collection. This includes designing experiments, conducting studies, and gathering empirical evidence^a^	- Experimental design - Instrument development - Data collection execution - Fieldwork/laboratory coordination - Data recording - Ethical compliance - Problem-solving
Methodology^a^	Developing and refining the research methods and procedures used for data collection and analysis. This ensures that the approaches are appropriate, reliable, and valid for addressing the research questions^a^	- Method selection - Protocol development - Study/protocol registration - Pilot testing - Analytical technique identification - Bias mitigation - Data management planning - Documentation
Data curation^a^	Managing, organising, and maintaining data throughout the project lifecycle. This involves ensuring data quality, integrity, and accessibility for analysis and future use^a^	- Data organisation - Data cleaning - Metadata creation - Data security - Access management - Regulatory compliance - Data preservation planning - Data sharing
Formal Analysis^a^	Applying statistical, computational, or qualitative analysis techniques to interpret the collected data and draw conclusions that address the research objectives^a^	- Statistical analysis - Model development - Qualitative analysis - Result interpretation - Hypothesis testing - Pattern identification - Analysis documentation - Code publication
Visualisation^a^	Creating graphical or visual representations of data and research findings to enhance understanding and effectively communicate results to various audiences^a^	- Chart and graph creation - Infographic development - Interactive visualisation - Figure preparation - Data mapping - Graphical design refinement - Visual accessibility compliance
Writing—original draft^a^	Composing the initial versions of all written project materials, including manuscripts, reports, and documentation that detail the research process and findings^a^	- Manuscript drafting - Report writing - Protocol compliance - Literature synthesis - Grant applications - Abstract and summary writing - Supplementary material preparation
Writing – Review & Editing^a^	Revising and refining written materials to improve clarity, coherence, and overall quality. This includes proofreading, incorporating feedback, and ensuring the content meets publication standards^a^	- Content editing - Proofreading - Feedback integration - Formatting compliance - Citation verification - Ethical review - Finalisation - Submission
Communication^b^	Disseminating research findings and project updates to both academic and non-academic audiences through various channels to enhance visibility and impact^b^	- Conference presentations - Journal publications - Media engagement - Social media outreach - Educational outreach - Stakeholder networking - Communication strategy development
Validation^a^ (checking)	Ensuring the accuracy, reliability, and validity of research findings through rigorous verification processes. This includes data checking, replication, and peer review to uphold research integrity^a^	- Data verification - Replication studies - Peer review solicitation - Methodological cross-validation - Quality control implementation - Error documentation - Robustness testing
Supervision^a^	Providing leadership, guidance, and support to the research team. This involves mentoring, overseeing progress, and ensuring that the project objectives are met effectively and efficiently^a^	- Expectation setting - Performance monitoring - Mentorship - Conflict resolution - Progress oversight - Resource allocation - Motivation and encouragement

^a^[35, 38, 62]

^b^[43]

### The current systems lack responsibility (R)

Responsibility, in this context, refers to clearly defining expectations at the outset of a project. If quest members do not know upfront what actions will qualify them for dragonslayer status, confusion and conflict will arise when it is time to assign titles later. In research, project contributors often lack clarity about what qualifies them for authorship or how their contributions will be weighted when assigning authorship positions [11, 37]. Establishing a prenuptial collaboration agreement to outline authorship rules has been suggested as a way to prevent disputes later on [8, 47, 68–70]. However, while the prevalence of such agreements is unclear, it is likely that only a small fraction of research teams currently adopt this practice.

### The current systems lack equity (E)

Without clear rules applied equally to everyone, some dragonslayers may receive undeserved credit or be overlooked entirely due to irrelevant factors. For example, the person with the most influence in the community may get credit even if they contributed very little or nothing [71]. Unfortunately, such behaviour is not unheard of in academia, and much has been written about gift authorship [65, 72–75]. On the other end of the spectrum of unequitable authorship behaviours, we have ghost authorship, where a person who significantly contributed is omitted from the author list [65, 73, 75–78]. Unsurprisingly, ghost authorship disproportionately affects early career researchers and those with less social capital [1, 79–81]. The current systems do not help alleviate either of these issues.

### The current systems lack autonomy (A)

If the quest leader is the only one who decides who gets the spoils and how much, nobody may dare to challenge their decisions. Further, if the eventual division of loot and glory is predetermined, dragonslayers cannot change their position as the quest progresses. In research projects, senior researchers hold the power (e.g., [11]) and established hierarchies or personal connections can determine authorship order [82]. Authorship order is also subject to conventional expectations and early promises that are expected to be upheld, often ignoring changing circumstances (e.g., shifts in team member involvement, the addition or removal of tasks, or changes in roles and responsibilities over the course of the project; 45). Solid evidence is needed to challenge authorship order. As such, there is no system for checks or balances, which can leave contributors unable to advocate for adjustments.

### The current systems lack transparency (T)

Our dragonslaying endeavour can become a tangle of myths and legends to even those who are part of the quest. Most quest members can only see what others are doing as long as their activities are within their own field of vision. This is also true for research where even if someone has an overview of everyone’s contributions, that information is not openly and continuously shared with all the members of a project. Contributors often do not know how authorship decisions are made throughout the project and they are ultimately only presented with the finalised list of authors when the research is written up. Contributors have no way of knowing, in detail, how much others contributed, and how they compare (e.g., [11]). Thus, it is here that the opaqueness of working separately becomes an issue.

## Dragon Kill Points

### The GREAT principles

Navigating our own experiences led us to consider and experiment with what an effective system for deciding authorship order might look like (e.g., [83–85]; Fig. 2). The principles we propose are designed to address the key shortcomings of the current systems outlined in the “The problem with the current status quo” section, namely, their lack of granularity, responsibility, equity, autonomy, and transparency. We propose that an effective system should (1) measure contributions with finer detail than current frameworks allow (Granularity); (2) ensure contributors know upfront what qualifies them for authorship and how their contributions will be measured (Responsibility); (3) apply rules consistently to everyone involved in a project (Equity); (4) enable contributors to change or challenge their position in the authorship list based on a record of contributions (Autonomy); and (5) keep contributors informed throughout the project about their record of contributions, potential authorship position, and how authorship decisions are being made (Transparency). By embedding these five GREAT principles into authorship practices, we can move toward more inclusive, fair, and accountable systems—especially important when evaluating the value of middle authorship positions [6, 8, 86].

### The framework and templates

To reduce the stress around authorship order decisions, we developed a simple and practical system called Dragon Kill Points, a term co-opted from video game culture [52]. Dragon Kill Points tracks authorship contributions in a way that, once in place, satisfies the five key principles we have outlined (Fig. 2). Dragon Kill Points ensures *granularity* by allowing detailed breakdowns of contributions (Table 1), *responsibility* by establishing rules upfront, *equity* through consistent application of these rules to all involved, *autonomy* because authorship position is rewarded based on documented contributions and can be challenged accordingly (Fig. 1), and *transparency* by keeping track of contributions and sharing it with all team members (Fig. 2), and the final record can be made publicly available alongside the author contributions statement (Fig. 2). Our experience so far has shown that Dragon Kill Points reduces conflicts over authorship by fostering an open, transparent dialogue surrounding contributionship and authorship order from the outset of the project.

We provide several free templates (CC BY) and a shiny application (CC BY) to make the process straightforward and accessible. These templates are designed to help minimise equity issues around accessibility and can be customised for a variety of project types. The templates are available in multiple formats (e.g., PDF, Excel, and Google spreadsheets) and have been uploaded to several platforms to increase their accessibility: supplemental materials, GitHub, Open Science Framework, figshare, and Google Drive (links provided in the data accessibility statement). Our templates cover the following types of projects to be adapted as needed: (1) fieldwork, (2) laboratory projects, (3) meta-science, (4) opinions and comments, (5) theoretical and modelling, and (6) a general template. For readers unfamiliar with Dragon Kill Points, an example of how these templates can be used is provided in Table S1, which illustrates the application of the “opinions and comments” template used for this publication (real-world examples are also available for fieldwork [84, 85] and meta-science [83]). This diversity ensures that regardless of the nature of your project, there is a framework in place to transparently and fairly assign authorship order for many fields.

## How to make Dragon Kill Points doable and accepted: guidelines for implementation

Implementing Dragon Kill Points effectively requires attention to the GREAT principles—granularity, responsibility, equity, autonomy, and transparency—that underpin its design (Fig. 1). A method of recording contributions should be created at a project’s outset (e.g., by adapting one of our templates). It should list each relevant task and its corresponding way of measuring and assigning weights, and can be refined iteratively as the project progresses (Fig. 2). Here is how to structure the system in a way that promotes wide adoption and smooth operation, regardless of team size or project type.

### Granularity (G): breaking down contributions with precision

The strength of Dragon Kill Points lies in its ability to provide granular detail when documenting contributions.

- *Task breakdown:* Contributions should be divided into distinct and manageable pieces [16, 43, 46]. This can range from major tasks (e.g., writing) to smaller but essential contributions (e.g., editing the manuscript), which could be further broken down to even finer detail (e.g., editing draft manuscript version 4.0).
- *Making the invisible visible:* Contributors can shed light on processes that might otherwise go unnoticed. This includes behind-the-scenes responsibilities, such as those conducted by supervisors or team leads that might otherwise never be seen by other team members.
- *Adjustable levels of detail:* While tasks can be broken down infinitely, it is important to strike a balance between detail and simplicity. Ensure that the process remains manageable without sacrificing the precision of contributions (e.g., yes/no option may be sufficient to record if someone edited the draft in any way rather than trying to capture the number of edits they made).
- *Weighted contributions:* Depending on the nature of the task, weights can be assigned to each contribution [11, 30]. This refers to how contributors’ efforts vary within a task, which is distinct from the weighting of the tasks themselves. For instance, more complex or time-intensive tasks can be given higher weights, ensuring that contributors receive credit proportional to their efforts (e.g., editing the whole manuscript draft may carry more weight than writing an abstract).

### Responsibility (R): setting expectations from the outset

For Dragon Kill Points to work effectively, responsibility means ensuring rules and expectations are explicit from the outset, so authorship order reflects the agreed framework rather than ad hoc decisions [60].

- *Initial discussions:* At the project’s inception, teams should discuss and agree on how contributions will be recorded and how points will be assigned. Everyone should know what is required for authorship and what factors (e.g., quality or quantity of work) will affect their position in the authorship order, including whether, and under what circumstances, contributors may change their authorship position and how such changes will be evaluated and agreed upon. This is also an opportunity to agree on set times to revisit discussions, whether at certain stages of the project or monthly check-ins.
- *Naming an arbiter:* Teams should designate a person that is taking the lead on making the template and keeping track of contributions. While individuals can be responsible for inputting their own contributions, having one person leading this task can limit data entry errors. The arbiter can also help navigate any disagreements should they arise (e.g., have the final say and lead a democratic vote). In practice, we have found that the person leading the project is often best suited to this role.
- *Meaning of order:* Contributors should have a clear understanding of how Dragon Kill Points is used to determine authorship position, and this should be clearly stated in the author contribution statement in the resulting manuscript. For example, contributions may decline with order (termed “sequence-determines-credit” by Tscharntke et al. [8]), be listed alphabetically for equal contributors ([58, 87]; termed “equal contribution” by Tscharntke et al. [8]), or emphasis may be placed on the first and last author positions (termed “first-last-author-emphasis” by Tscharntke et al. [8]). Combinations of these approaches can be used too (e.g., “first-last-author-emphasis” and “sequence-determines-credit” for the middle authors).
- *Continuous dialogue:* Dragon Kill Points should not be a static process. It can and must change with different contexts and needs. Revisiting the rules and expectations throughout the project encourages self-regulation and ensures that contributors are aware of any changes. Revisions to agreed authorship positions, including adjustments due to unplanned additional contributions, should be discussed and confirmed collectively to ensure any changes reflect shared understanding rather than unilateral action. This proactive approach leads to smoother collaboration and avoids conflicts related to not only missed or underestimated contributions but also the addition of previously unplanned contributors.
- *Field-specific flexibility:* When needed, authors can opt for not using Dragon Kill Points for specific positions (e.g., last author, corresponding author, etc.). This will allow for better integration of this system with current conventions across multiple disciplines [6, 11, 20].

A responsible framework ensures that all contributors are on the same page from the outset, preventing surprises and animosity when the project concludes.

### Equity (E): applying the rules consistently

Equity is critical to the success of Dragon Kill Points. The system should be designed in a way that ensures fair treatment for all contributors (i.e., creating equity through equality).

- *Consistent application of rules:* The same set of guidelines should apply to everyone, regardless of their position, experience, or reputation. Equity should be prioritised by focusing on the quality and impact of contributions rather than arbitrary metrics (e.g., previous work history, status, etc. [88]). The distinction between equity and equality can be difficult when advocating for a system that applies the same criteria to everyone. The distinction is in whether the established hierarchy is maintained as a rule rather than as happenstance (e.g., [11]).
- *Careful attention to categories *(Table 1): Care should be taken not to favour or discriminate against certain individuals unintentionally when creating rules for measuring contributions [67, 89–92]. Metrics that could lead to manipulation or bias (e.g., seniority or number of hours worked on a task) should be avoided [88]. Instead, the focus should be on quantifying the contributions, not the contributors themselves.
- *Moving towards objectivity:* When measuring contributions, avoid scoring contributions with vague or subjective metrics. For example, the International Committee of Medical Journal Editors [93] suggests including authors that have made a “significant contribution”, which is open to interpretation. Likewise, Martins et al. [11] advocate for rating contributions along a spectrum from “major” to “minor”. These practices risk increasing inequities because of the subjective nature of rating [94]. Instead, we advocate for using less ambiguous metrics (e.g., counting the number of samples they measured), while recognising that certain tasks may inherently carry more weight than others (e.g., writing the discussion section versus the methods section).
- *Gaming the system:* Dragon Kill Points is not immune to manipulation, but does allow for it to be more readily detected. Care should be taken to monitor and disincentivise such opportunities. Self-reporting, as advocated by Martins et al. [11], and maximising Dragon Kill Points through minimal effort are the easiest weaknesses to this system. Version histories of data and files can aid in regulating the first (“Transparency (T): ensuring an open process” section), while the second needs to be curtailed through well-considered metrics.

Our expectation is not that Dragon Kill Points will eliminate all cases of inequitable authorship, but that by requiring regular, specific documentation of contributions in a shared and visible record, it raises the cost of dishonesty, provides evidence to support disputes, and promotes a culture in which equity is easier to uphold.

### Autonomy (A): empowering contributors to manage their contributions

Dragon Kill Points allows contributors to maintain some level of autonomy over their authorship position by continuously engaging in the system throughout the project’s lifecycle.

- *Adjustable contributions:* Contributors should be able to alter their authorship rank through their contributions. If Dragon Kill Points are used to determine authorship order, authors should have opportunities to “level up” their rank (e.g., by processing additional laboratory samples or drafting figures for a final publication). As noted in our discussion of weighting (“Equity (E): applying the rules consistently” section), certain tasks may inherently carry more weight than others. However, as a general principle, no single task should carry disproportionate weight such that exclusion from participating in it alone would override contributing meaningfully to the project as a whole [11].
- *Supporting evidence:* Contributors should be able to challenge their authorship position based on the recorded evidence [24]. For example, if someone has been omitted from the authorship list but has recorded many contributions, the evidence can be used to claim the authorship, or to seek support for such a claim from team members.
- *Fixed-order disciplines:* In some disciplines, authorship order is standardised as a rule (e.g., alphabetical; reviewed in [13]). This predetermined order can offer a clear vantage point for applying Dragon Kill Points, by making the underlying contributions more straightforward for readers and assessors to interpret. In such cases, Dragon Kill Points can still be used to argue for authorship. Tracking contributions may then fill a different role instead of determining authorship order, such as communicating which author should be contacted to discuss specific details of a project or for evaluation by funding committees.

This dynamic system promotes engagement and ensures that contributors feel they have the autonomy to adjust their role as needed, making the process more reflective of actual *contributions.*

### Transparency (T): ensuring an open process

The transparency of Dragon Kill Points means all contributions and decisions are visible, traceable, and accessible to every team member.

- *“Pre-registration”*: Dragon Kill Points should be set up at the start of a project, with categories, tasks, and any weighting agreed upon in advance and recorded in a shared, time-stamped document (“Granularity (G): breaking down contributions with precision” and “Responsibility (R): setting expectations from the outset” sections). Records should be maintained in a format with automatic version control (e.g., Google Sheets, GitHub, OSF) so that all edits are visible to the team, and entries should be updated regularly (e.g., monthly) as contributions occur. This creates a transparent, ongoing record that discourages retroactive adjustments intended to justify a predetermined authorship list. Dragon Kill Points is conceptually similar to pre-registration in that criteria and weights are agreed upon before work begins, but unlike pre-registration, authors’ contributions as recorded by Dragon Kill Points are updated continuously.
- *Shared access:* An up-to-date Dragon Kill Points table with contributions recorded should be available to everyone throughout the project. This allows contributors to track their own progress as well as others’, facilitating discussions on authorship before issues arise [4]. Whenever possible, each entry should be supported with verifiable evidence, such as links to shared documents or datasets, commits for code, or timestamps for completed tasks.
- *Clear communication:* Any changes made to the contributions or rules should be discussed, documented, and shared with the team. The process of logging and evaluating contributions should remain open, ensuring no one is left out of key decisions (e.g., sharing documentation along with progress emails to all team members).
- *Visibility:* The detailed tables of contributions should be shared with the broader research community after the project is completed (e.g., in the supplementary materials). Including links to supporting records (where possible) in the final shared table further enhances credibility and encourages transparency across multiple levels of collaboration.

A transparent system fosters trust and accountability among team members along with the scientific community [95]. Contributors can be confident that their efforts will be publicly recognised, and they will have a clear understanding of where they should stand in the authorship list.

## Practical considerations for adopting Dragon Kill Points

### Keep it simple

Dragon Kill Points works best when it is straightforward to use. Google Sheets, GitHub, and OSF allow for automatic version control and are no- to low-cost, accessible, and free from unnecessary software or technical barriers (e.g., [46, 63]). Dragon Kill Points scales easily from small team teams (≥ 2 people) to larger collaborations and can be adapted for many project types, including fieldwork, laboratory projects, reviews, opinion pieces, and theoretical work, as examples, using our free, ready-to-use templates or shiny application (see “Data Accessibility”). While at its core, Dragon Kill Points is designed to facilitate the determination of authorship order, field-specific customs may dictate that authorship be based on other criteria, such as alphabetically, regardless of contribution [58, 87]. In such cases, Dragon Kill Points would not be employed to determine order, but the underlying data that it contains (i.e., who did what and to what extent) may still warrant inclusion and monitoring.

### Time investment versus payoff

A major concern with implementing any new system is the perceived time investment. However, significant, often unmeasured, time is already spent on people management and authorship discussions throughout projects, even if we do not consciously track it. Setting up Dragon Kill Points takes some upfront work, mainly creating the template and selecting contribution categories (Table 1), but this is greatly reduced with our ready-to-use templates or shiny application (see “Data Accessibility”). Once established, upkeep is minimal, averaging about one minute per person per update. This small investment pays off by reducing the cognitive burden and stress of navigating difficult social dynamics [4]. By clearly defining who qualifies as an author, their roles, and (if used) their authorship order, Dragon Kill Points reduces ambiguity, minimises misunderstandings, and helps prevent disputes.

### Breaking the first rule: talking about authorship

Authorship disputes are already common at the graduate level, with higher rates reported by historically marginalised groups [96], making early and open conversations critical. The best time to introduce Dragon Kill Points, thus, is as early as possible. Clear expectations from the outset make adoption easier, and those in leadership roles are often best positioned to champion the approach, although early career researchers can also lead implementation with mentor support. A gentle entry point could be to suggest this paper for a journal club discussion, sharing Fig. 1 for a light-hearted entry point, or by having team members reflect on tasks from past projects that they felt were undervalued, then using these as a basis for customising the Dragon Kill Points template to fit the team’s specific needs.

It can be effective to frame Dragon Kill Points not only as a tool for improving collaboration and tracking contributions, but also as a way to strengthen *psychological safety* and mentoring, which are known to support effective shared leadership and collaboration in research networks [18]. Work on psychological safety shows that hierarchy and job security shape who actually feels safe to raise a concern [97], while people are known to weigh the risk of punishment before speaking, especially when power dynamics are uneven [98]. These dynamics mean that the loudest voices in a team may also be the safest voices, leaving others without a constructive way to voice concerns.

## Let’s slay the dragon together!

A social shift, aided by a systemic shift, is called for in academic authorship practices [18, 45, 48, 51, 69, 70, 99, 100]. Implementing structured frameworks like Dragon Kill Points can help normalise these conversations—conversations that are often difficult and awkward. When teams have clear, transparent guidelines to track and measure contributions, it becomes easier for researchers to advocate for their work to be acknowledged [95]. Although this approach requires some upfront effort to set expectations, these discussions should be occurring at the project’s outset anyway [4, 23, 47, 69, 101]. Our projects—our quests—should begin with open dialogue to avoid authorship decisions being made after the fact or against the evidence.

While Dragon Kill Points is tailored to journal article authorship, its potential reaches far beyond. Authorship disputes arise across a variety of media, including conference proceedings, government reports, software packages, undergraduate group assignments with contribution-based grades, reagents, books, and even movie credits [45, 48]. In all these areas, contributors may go unrecognised or be placed in positions that do not reflect their actual input. It would even allow authors to document their use of large language models, including whether these tools warrant authorship.

Of course, Dragon Kill Points may not be perfectly suited to every scenario – projects with only one contributor or massive collaborations with hundreds of participants will have different needs. However, for most collaborative teams, particularly those of three or more people, Dragon Kill Points offers a GREAT method for managing contributions if adopted transparently and consistently.

People management is like data management; you need to know your workflow and elements beforehand. In both cases, if you do not do it properly, you are either losing data or people. Dragon Kill Points is a tool designed to simplify and normalise the authorship conversation, ensure fairness, and foster an environment where contributions are visible and trusted. Let’s slay the dragon together—without turning on each other throughout the quest.

## Supplementary Information

Supplementary Material 1: Table S1. Contributions are scored as 1 (yes) or 0 (no). Total counts are used when a non-binary system is not appropriate.

## Data Availability

We have included all templates in our supplemental material. We have additionally made all templates available through our dedicated GitHub repository (https://github.com/martinig/dragon-kill-points), including our shiny application (https://github.com/szymekdr/dragonkillpoints_app), Center for Open Science (https://osf.io/58qh4/?view_only=e69ab7df51394b00a4d9312d85603b3f), figshare (10.6084/m9.figshare.28405985.v1), and Google Drive (https://drive.google.com/drive/folders/1V8pxeQiAR7LJdyQ7Iu5v7Y8k7VNGyQe1?usp=sharing).
