# Supplementary material for: Dragon Kill Points: applying a transparent working template to relieve authorship stress
Source: BMC Biol. 2026 Jan 29;24:48. doi: 10.1186/s12915-026-02521-x (PMC12924437; doi:10.1186/s12915-026-02521-x)
Supplement: Supplementary file 1 — Supplementary Material 1: Table S1. Contributions are scored as 1 (yes) or 0 (no). Total counts are used when a non-binary system is not appropriate. [file 12915_2026_2521_MOESM1_ESM.pdf]

Supplemental Material

Table S1. Contributions are scored as 1 (yes) or 0 (no). Total counts are used when a non-binary system is not appropriate.

**name** (listed alphabetically by last name)	**joined project (mdy)**	**left project (mdy)**	**conceptualization**	**templates**	**literature search and literature summary**	**update templates after literature review**	**open access spreadsheet formatting (PDF, Excel, Google spreadsheets)**	**open access platforms (OSF, Github, figshare, google drive, and website)**	**dragon figure (5 pts)**	**dragon figure feedback (3 pts)**	**template figure (5 pts)**	**template figure feedback (3 pts)**	**table (5 pts)**	**table feedback**	**glossary**	**glossary feedback**
Burk, Spenser L. P.	11/6/2023	NA	1	0	1	0	0	0	5	3	0	2	1	0	0	0
Burke, Samantha	11/20/2023	12/10/2024	0	0	1	NA	NA	NA	NA	NA	NA	NA	NA	NA	NA	NA
Ceccacci, Alberto	12/10/2024	NA	NA	NA	NA	0	0	0	NA	0	NA	1	NA	0	NA	0
Chhen, Aimee	12/10/2024	NA	NA	NA	NA	0	1	0	0	1	0	1	0	0	0	0
Cincotta, Joe	not participating	NA	NA	NA	NA	NA	NA	NA	NA	NA	NA	NA	NA	NA	NA	NA
Cuadros, Sandra	12/10/2024	NA	NA	NA	NA	0	0	0	0	1	0	1	0	0	0	0
de Jong, Julia	12/11/2024	NA	NA	NA	NA	0	0	0	5	0	0	0	0	0	0	0
Drobniak, Szymon M.	11/15/2023	NA	0	2	1	0	0	0	5	1	5	1	0	1	0	0
Gibson, Matthew J.	11/5/2023	12/10/2024	NA	NA	NA	NA	NA	NA	NA	NA	NA	NA	NA	NA	NA	NA
Lagisz, Malgorzata	11/3/2023	NA	1	1	1	0	0	0	0	3	0	2	1	1	0	1
Martinig, April Robin	11/3/2023	NA	1	4	1	6	1	1	5	2	5	3	1	1	1	0
Morrison, Kyle	11/5/2023	NA	0	1	1	0	0	0	0	0	0	0	5	0	0	0
Mizuno, Ayumi	11/5/2023	NA	0	0	1	0	0	0	0	0	0	1	0	1	0	0
Nakagawa, Shinichi	11/3/2023	NA	1	0	1	0	0	0	0	1	0	1	0	0	0	0
Perry, Isabella	12/10/2024	NA	NA	NA	NA	6	0	0	0	2	0	1	2	0	0	0
Petersohn, Megan	12/17/2024	NA	NA	NA	NA	0	5	0	0	0	0	1	0	1	0	0
Pollo, Pietro	11/5/2023	NA	0	0	1	0	0	0	0	2	0	1	0	0	0	0
Pottier, Patrice	11/5/2023	NA	0	0	1	0	0	0	0	1	0	1	0	1	0	1
Ricolfi, Lorenzo	11/5/2023	NA	0	0	1	0	0	0	0	1	0	1	0	0	0	1
Tam, Jess	11/8/2023	NA	0	0	0	0	0	1	0	1	0	1	0	0	0	0
Williams, Coralie	11/5/2023	NA	0	0	1	0	0	0	0	0	0	1	0	0	0	1
Yang, Yefeng	11/5/2023	NA	0	0	1	0	0	0	0	0	0	0	0	1	0	0

**name** (listed alphabetically by last name)	**draft 1 writing (5 pts)**	**draft 1 feedback (5 pts)**	**draft 2 writing (5 pts)**	**draft 2 feedback (5 pts)**	**draft 3 writing (5 pts)**	**draft 3 feedback (5 pts)**	**draft 4 writing (5 pts)**	**draft 4 feedback (5 pts)**	**draft 5 writing (5 pts)**	**draft 5 feedback (5 pts)**	**draft 6 writing (5 pts)**	**draft 6 feedback (5 pts)**
Burk, Spenser L. P.	5	0	5	0	2	0	4	0	3	2	1	4
Burke, Samantha	NA	NA	NA	NA	NA	NA	NA	NA	NA	NA	NA	NA
Ceccacci, Alberto	NA	NA	NA	NA	NA	NA	NA	NA	NA	NA	NA	0
Chhen, Aimee	NA	NA	NA	NA	NA	NA	NA	NA	NA	NA	NA	5
Cincotta, Joe	NA	NA	NA	NA	NA	NA	NA	NA	NA	NA	NA	NA
Cuadros, Sandra	NA	NA	NA	NA	NA	NA	NA	NA	NA	NA	NA	1
de Jong, Julia	NA	NA	NA	NA	NA	NA	NA	NA	NA	NA	NA	NA
Drobniak, Szymon M.	0	0	0	0	0	0	0	0	0	0	0	4
Gibson, Matthew J.	NA	NA	NA	NA	NA	NA	NA	NA	NA	NA	NA	NA
Lagisz, Malgorzata	1	4	2	2	0	5	4	1	2	3	0	0
Martinig, April Robin	5	0	5	0	2	0	5	0	3	0	3	0
Morrison, Kyle	0	0	0	0	0	0	0	0	0	0	0	5
Mizuno, Ayumi	0	0	0	0	0	0	0	0	0	0	0	5
Nakagawa, Shinichi	0	1	0	0	0	0	0	0	0	4	0	0
Perry, Isabella	NA	NA	NA	NA	NA	NA	NA	NA	NA	NA	NA	2
Petersohn, Megan	NA	NA	NA	NA	NA	NA	NA	NA	NA	NA	NA	4
Pollo, Pietro	0	0	0	0	0	0	0	0	0	0	0	5
Pottier, Patrice	0	0	0	0	0	0	0	0	0	0	0	5
Ricolfi, Lorenzo	0	0	0	0	0	0	0	0	0	0	0	5
Tam, Jess	0	0	0	0	0	0	0	0	0	0	0	5
Williams, Coralie	0	0	0	0	0	0	0	0	0	0	0	5
Yang, Yefeng	0	0	0	0	0	0	0	0	0	0	0	5

**name** (listed alphabetically by last name)	**final draft writing (5 pts)**	**final draft feedback (1 pt)**	**MS formatting for submission**	**cover letter for submission**	**website for dragon kill points**	**authorship order finalized**	**approval to submit**	**sum contribution scores**	**author order (*indicates equal authorship)**
Burk, Spenser L. P.	5	1	1	0	0	NA	1	46	2
Burke, Samantha	NA	NA	NA	NA	NA	NA	NA	1	NA
Ceccacci, Alberto	2	1	0	0	0	NA	1	4	17
Chhen, Aimee	NA	0	0	0	0	NA	1	8	12*
Cincotta, Joe	NA	NA	NA	NA	NA	NA	NA	0	NA
Cuadros, Sandra	NA	0	0	0	0	NA	1	3	18
de Jong, Julia	NA	0	0	0	0	NA	1	5	16
Drobniak, Szymon M.	0	0	0	0	0	NA	1	20	3
Gibson, Matthew J.	NA	NA	NA	NA	NA	NA	NA	0	NA
Lagisz, Malgorzata	3	1	0	0	0	NA	1	39	19
Martinig, April Robin	5	0	1	1	0	NA	1	62	1
Morrison, Kyle	0	1	0	0	0	NA	1	13	5
Mizuno, Ayumi	0	0	0	0	0	NA	1	8	12*
Nakagawa, Shinichi	0	1	0	0	0	NA	1	10	8*
Perry, Isabella	NA	1	0	0	0	NA	1	14	4
Petersohn, Megan	NA	0	0	0	0	NA	1	11	6*
Pollo, Pietro	0	0	1	0	0	NA	1	10	8*
Pottier, Patrice	0	1	0	0	0	NA	1	11	6*
Ricolfi, Lorenzo	0	1	0	0	0	NA	1	10	8*
Tam, Jess	0	0	0	0	5	NA	1	8	12*
Williams, Coralie	0	1	0	0	0	NA	1	9	11
Yang, Yefeng	0	0	0	0	0	NA	1	7	15
